# Bureau of Animal Industry

**Published:** 1903-03

**Authors:** 


### BUREAU OF ANIMAL INDUSTRY.

The many changes of advanced salaries to those on the veterinary staff as announced in this number is a gratifying evidence of the Department's disposition to retain the best services possible in this work. The marked advance in all salaries accorded to veterinarians over those of five or more years ago, marks the progress and appreciation of the work so well performed for the people generally, that we look forward to this department as the one that will lead in true scientific progress and development.

